# Reversals in Movement Direction in Locomotor Interception of Uniformly Moving Targets

**DOI:** 10.3389/fpsyg.2021.562806

**Published:** 2021-02-18

**Authors:** Gwenaelle Ceyte, Remy Casanova, Reinoud J. Bootsma

**Affiliations:** Institut des Sciences du Mouvement, Aix-Marseille Université, CNRS, Marseille, France

**Keywords:** interception, steering, locomotion, constant bearing strategy, reversal movements, first-order information, fractional order

## Abstract

Here we studied how participants steer to intercept uniformly moving targets in a virtual driving task. We tested the hypothesis that locomotor interception behavior cannot fully be explained by a strategy of nulling rate of change in pertinent agent-target relations such as the target-heading angle or target’s bearing angle. In line with a previously reported observation and model simulations, we found that, under specific combinations of initial target eccentricity and target motion direction, locomotor paths revealed reversals in movement direction. This phenomenon is not compatible with unique reliance on first-order (i.e., rate-of-change based) information in the case of uniformly moving targets. We also found that, as expected, such reversals in movement direction were not observed consistently over all trials of the same experimental condition: their presence depended on the timing of the first steering action effected by the participant, with only early steering actions leading to reversals in movement direction. These particular characteristics of the direction-reversal phenomenon demonstrated here for a locomotor interception-by-steering task correspond to those reported for lateral manual interception. Together, these findings suggest that control strategies operating in manual and locomotor interception may at least share certain characteristics.

## Introduction

**FIGURE 1:**
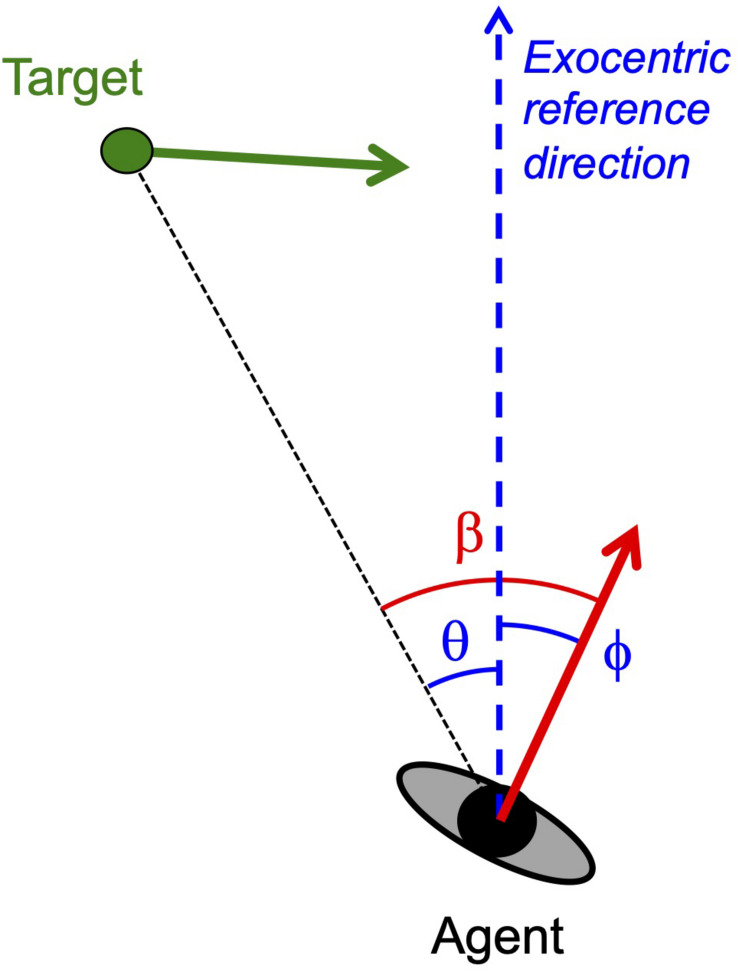
Definition of variables in a plan view of an agent moving through an environment containing a target moving in the same plane. Instantaneous velocity vectors are represented by arrows (red for agent, green for target). Agent heading ϕ and target bearing θ are defined with respect to an exocentric reference direction (dashed blue line). Target-heading angle β is defined by the eccentricity of the target with respect to the agent’s direction of locomotion so that β = ϕ − θ. Uniform target movement is defined by invariance of the target’s velocity vector in both orientation and magnitude.

More often than not conclusions about the information used in locomotor interception^1^ are based on the global correspondence of the behavioral patterns observed to one of several heuristically defined interception strategies (for examples see Kane and Zamani, 2014; Gonzalez-Bellido et al., 2016). Commonly evoked heuristics include continuously moving in the current direction of the target which corresponds to moving so as to maintain target-heading angle β at zero (*classical pursuit strategy*), continuously moving so as to maintain target-heading angle β constant at a non-zero value (*constant target-heading angle or CTHA strategy*), and continuously moving so as to maintain the target’s bearing angle θ constant at a non-zero value (*constant bearing or CB strategy*); see Figure 1 for definitions of pertinent angles. However, attractive such a global correspondence approach to identifying the operative interception strategy may appear to be, it is important to realize that the finding of relative constancy (e.g., β close to constant) over the course of interception only provides circumstantial evidence in favor of the associated strategy, as it remains unclear how this state of constancy came to be in the first place and how it may be restored following perturbation. Direct evidence for any interception strategy therefore requires defining such strategies not in terms of rule-of-thumb heuristics but in terms of dynamics, that is, in terms of how the system evolves over time toward a steady-state regime (Bootsma et al., 2016). In dynamical terms, maintaining target-heading angle at zero translates into the agent seeking to null β, while maintaining β or θ constant translates into the agent seeking to null changes in, respectively, β or θ. Adopting such a dynamical perspective leads one to focus on transients rather than steady-state regimes.

In this framework, the present contribution elaborates on the observation of an unexpected transient behavior reported by Fajen and Warren (2004) in a study of locomotor interception of uniformly moving targets: under specific initial conditions, freely walking participants were found to demonstrate reversals in movement direction over the course of their interceptive actions. In order to situate this unexpected transient behavior within both Fajen and Warren’s (2004) larger set of results and Fajen and Warren’s (2007) extensive analysis of the underlying dynamics, we first briefly recall the experimental setting and results.

In Fajen and Warren’s (2004) experimental protocol targets either appeared directly in front (CENTER condition) or 25° to the left (SIDE condition) of the walking participant. Moving rightward^2^ at constant speed, targets followed rectilinear trajectories either oriented perpendicular to the participant’s initial locomotor direction (Cross direction), approaching the participant at an angle of 30° to the perpendicular (Approach direction) or retreating at an angle of 30° to the perpendicular (Retreat direction). Under the SIDE condition, where the target started from a position to the left, target and participant walking speeds resulted for the Approach target direction in interception occurring on the left of the participant’s initial locomotor direction. For the Cross and Retreat target directions, on the other hand, these same speeds resulted in interception occurring on the right of the participant’s initial locomotor direction. Under the CENTER condition targets were of course always intercepted on the right of the participant’s initial locomotor direction. Most likely due to gait cycle-induced variability in the individual trials, Fajen and Warren (2004) only presented overall average data patterns.

Fajen and Warren (2007) concluded that the overall shapes of the observed locomotor paths were not compatible with a *classical pursuit strategy* of nulling β (i.e., striving to bring β to zero). Behavior observed under the CENTER condition led Fajen and Warren (2007) to also invalidate the operation of a *constant target-heading strategy* based on nulling changes in β (i.e., striving to maintain β constant by dβ/dt-nulling). Initial conditions here placed the participant in a situation of lagging the target, due to its immediate outward movement. With target-heading angle β thus initially widening, a dβ/dt-nulling strategy would merely counter this growth, leading the participant to continue to lag the target. This scenario is not compatible with the straightening out of locomotor paths observed for the Cross and Retreat target directions under the CENTER condition.

As demonstrated by numerical simulation, a dθ/dt-nulling model on the other hand effectively captured almost all the characteristics of the locomotor paths for all target directions under both the CENTER and SIDE conditions. The single locomotor path characteristic that such a dynamical instantiation of the *constant bearing strategy* did not account for was the “slight S-shaped bend” (Fajen and Warren, 2007, p. 311) in the locomotor paths observed under the SIDE condition for the Cross and Retreat target directions. This same characteristic was described in their earlier experimental report as “participants turn smoothly toward the target, and (…) subsequently reverse back again to track the target motion” (Fajen and Warren, 2004, p. 697). Because for uniformly moving targets this direction-reversal phenomenon implies that participants did not uniquely rely on any kind of first-order (i.e., rate-of-change based) information^3^, it is on this transient behavior characteristic during the interception of uniformly moving targets that we focus in the present contribution.

We note that reversals in movement direction have also been reported in manual interception of uniformly moving targets (Montagne et al., 1999), indicating that control strategies operating in manual and locomotor interception may at least share certain characteristics. Interestingly, in manual interception the direction-reversal effect reported by Montagne et al. (1999) was not consistently present in all relevant experimental trials. This irregularity has in fact been argued to question the reliability of Montagne et al. (1999)’s findings (Dessing et al., 2002; Arzamarski et al., 2007). Yet, Bootsma et al. (2016) recently reported similar irregular observations of reversals in movement direction for participants intercepting targets following curving trajectories in a lateral locomotor interception setting. As this latter finding concerned non-uniformly moving targets, it is not directly comparable to those of either Montagne et al. (1999) or Fajen and Warren (2004). Relevant for the present purposes, however, Bootsma et al. (2016) demonstrated that the co-occurrence, over repeated trials of a same target trajectory condition, of both trials with and trials without a reversal in movement direction could be linked to differences in the timing of movement initiation. Specifically, in trials in which a reversal in movement direction was observed the interception movement was typically initiated early on, while in trials in which such a reversal in movement direction was not observed the interception movement was typically initiated later on.

Fajen and Warren (2007) suggested that the presence of the S-shaped bend (i.e., reversal in movement direction) in the locomotor paths observed under the SIDE condition for the Cross and Retreat target directions resulted from a latency to detect and respond to target motion, presumably related to participants having to parse the optic flow into self-motion and target motion components (Warren and Rushton, 2009). With the target initially perceived as stationary, participants would first steer toward it before reversing direction when target motion was detected and integrated. Operationally, they included this latency into their model in the form of a [0,1] interval-valued coefficient that modulates the influence of the component of dθ/dt due to target velocity as a sigmoidal function of time. The dθ/dt component due to self-motion was left unaffected. Latency duration (i.e., time until full integration of target motion) was estimated at 0.5 s for a structured environment including a static background. Congruent with the above-described findings of Bootsma et al. (2016), implementing Fajen and Warren’s (2007) dθ/dt-nulling model with such a latency function incorporated revealed that immediate initiation of steering gave rise to the observed reversals in movement direction, while delayed initiation did not (because the dθ/dt latency modulation was no longer operative).

In line with the overall goal of our research program into the information used for interceptive actions, in the present study we replicated and extended the experimental target motion conditions of Fajen and Warren (2004) in order to examine whether reversals in movement direction could be identified at the level of individual trials in an interception-by-steering task with uniformly moving targets, and if so, whether they occurred systematically (i.e., under certain conditions, but not others) and irregularly (i.e., on some but not all trials of a given condition). We included two (rather than only one) types of SIDE condition, as model simulations predicted that, for larger initial target eccentricities, comparable ranges of variation in the moment of initiation of the first steering action would lead to a larger proportion of trials with a reversal in movement direction as well as larger pre-reversal excursion amplitudes in those trials.

## Materials and Methods

### Participants

Nine students from Aix-Marseille University (four women and five men, mean age 24.3 ± 2.0 years) voluntarily took part in the experiment. Participants provided written consent prior to participation. The study was approved by Aix-Marseille University’s Ethics Committee and conducted according to University regulations and the Declaration of Helsinki.

### Task and Procedure

The experiment took place in a large virtual reality facility^4^ comprising four projection surfaces, each served by two projectors: a 3 × 3-m floor and three 4-m high × 3-m wide walls. The sidewalls were set at 90° angles with respect to the front wall. A basic driving simulator, comprising a seat, a set of (here non-operative) pedals and a steering wheel, was positioned in the middle of the floor surface. Stereopsis was ensured with passive Infitec filter technology. Participants’ stereo glasses were equipped with a configuration of reflective markers. An eight-camera Advanced Realtime Tracking (ART, Weilheim, Germany) optical system enabled real-time motion capture of head position. The visual scene was refreshed at 60 Hz, taking into account the position and orientation of the participant’s head relative to the virtual environment.

The visual scene consisted of a large grass-like textured flat plain bordered by distant mountains. The seated participant was instructed that on each trial the goal was to steer the “car” so as to intercept a horizontally moving yellow cylinder (2-m radius, 3-m high) by driving through it. Prior to trial onset, the participant, moving at a constant horizontal speed of 20 m/s, was to steer toward and subsequently align locomotor direction with a yellow line on the plain’s ground. Alignment was considered as accomplished when the car’s center (i.e., seat) attained a lateral distance of less than 3 cm with respect to the middle of the line while moving in a direction deviating less than 0.1° from the line orientation. This quite demanding requirement led participants to only minimally turn the steering wheel in the last stages of alignment. Once the alignment criteria were met, the yellow line disappeared and, at the same time, a red portal appeared 40 m ahead. Participants instructions stipulated that they should refrain from further steering from the moment onward that correct alignment had been achieved and until they passed through the portal. In fact, without the participants being aware of this, during that period the steering wheel was deactivated with wheel orientation recalibrated to zero, so that when the participant crossed the portal and the target appeared, they moved such that both ϕ = 0° and dϕ/dt = 0°/s. A trial ended when the participant came within the target’s circumference (successful interception) or when the participant reached a position further than 20 m in depth (*Z*-axis) beyond the current target position (missed trial).

**FIGURE 2:**
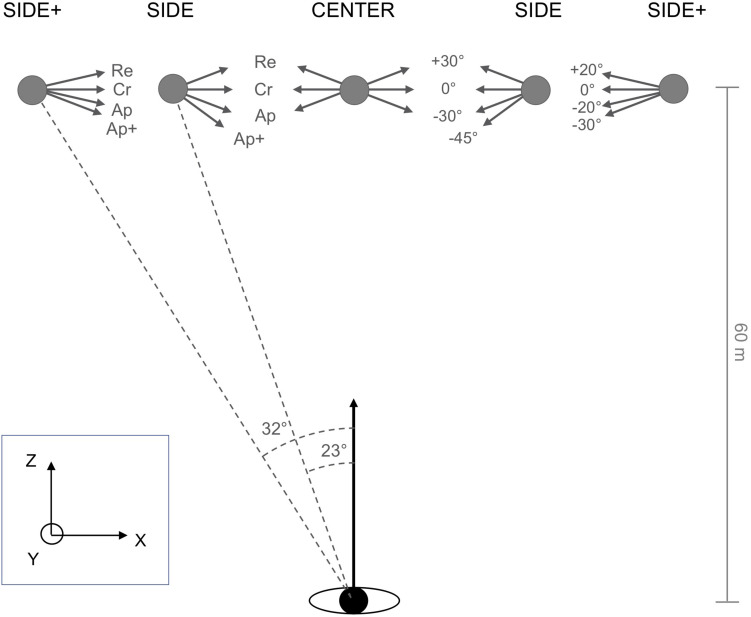
Plan view of the initial conditions of the experiment. The moving target appeared at CENTER (0°), SIDE (23°), or SIDE+ (32°) eccentricities with respect to the participant’s initial heading direction. Under CENTER and SIDE eccentricity conditions, the target moved at 10 m/s along Cross (Cr, 0°), Retreat (Re, +30°), and Approach (Ap, –30°) directions, with an additional Approach+ (Ap+, –45°) direction added under the SIDE eccentricity condition. Under the SIDE+ eccentricity condition, the target moved at 15 m/s along Cross (Cr, 0°), Retreat (Re, +20°), Approach (Ap, –20°), or Approach+ (Ap+, –30°) directions.

In the experimental trials targets were initially positioned at *Z* = 60 m and *X* = 0.00 m, −25.35 m or −38.08 m (see Figure 2), with initial participant position serving as the origin of the reference frame. Targets could thus appear at eccentricities of 0° (CENTER), 23° (SIDE) to the left, and 32° (SIDE+) to the left of the participant’s initial locomotor direction. Similar to Fajen and Warren’s (2004) protocol, under the CENTER and SIDE conditions targets moved at half the participant speed, here 10 m/s. Retreat, Cross, and Approach target directions (oriented, respectively, +30°, 0°, and −30° relative to the perpendicular with respect to the participant’s initial locomotor direction) were complemented with a −45° Approach+ target direction, so as to balance interceptions on the left and right side under the SIDE condition and to regularly confront participants with target trajectories requiring a rapid response. Under the SIDE+ condition, target speed was set to 15 m/s and target directions to +20° (Retreat), 0° (Cross), −20° (Approach), and −30° (Approach+), expected to give rise to interceptions locations comparable to those under the SIDE condition for all but the Retreat target direction. All these initial conditions were also mirrored left/right and the data were subsequently collapsed.

The full set of 22 experimental conditions was presented in randomized order within a block of trials. Participants performed five blocks, for a total of 110 trials (10 per mirror collapsed condition) and were invited to take a short break between blocks. Prior to the experiment proper participants performed 12 familiarization trials with stationary targets.

### Data Acquisition and Analysis

Participant position (X, Z) and orientation (ϕ) data were sampled at 100 Hz. These time series were subsequently filtered using a second-order Butterworth filter with a cut-off frequency of 8 Hz and collapsed over mirror conditions. For each trial, the closest distance to the target was calculated, corresponding to first contact with the target’s circumference for successful interception trials and minimal Euclidean distance from the target’s circumference for missed trials. Time to closest distance was defined as the time from onset of the trial until the moment the closest distance was reached. Bearing angle θ was derived at each time step using Matlab function atan2 for the quotient of target-agent distance in depth (Z_t_−Z_a_) over lateral target-agent distance (X_t_−X_a_). Time derivatives of ϕ and θ were obtained using the Euler method.

The moment of initiation of the first steering action was determined for each trial as the time after trial onset at which the participant’s rate of change in locomotor direction (dϕ/dt) exceeded 4°/s. A reversal in movement direction was defined as a lateral excursion of more than 0.05 m following initiation of the first steering action accompanied by a subsequent sign change in dϕ/dt leading to a movement in the opposite lateral direction. Pre-reversal excursion amplitude was defined as the largest initial lateral displacement before movement direction was reversed.

As Approach+ target directions were only present under the SIDE and SIDE+ eccentricity conditions, overall statistical analyses of success rate and moment of initiation of the first steering action were performed in two steps. First, a repeated-measures ANOVA with factors Eccentricity (CENTER, SIDE, SIDE+) and Direction (Approach, Cross, Retreat) was used to evaluate overall differences, using *post hoc* Newman–Keuls tests to clarify pairwise differences for significant effects. Second, a repeated-measures ANOVA with factors Eccentricity (SIDE, SIDE+) and Direction (Approach+, Approach, Cross, Retreat) was used to confirm the former analysis and pinpoint potential differences between Approach and Approach+ directions using *post hoc* Newman–Keuls tests where appropriate. More localized effects were evaluated using Chi-squared tests for frequency comparisons and Student *t*-tests for pre-reversal excursion amplitudes in trials with a reversal in movement direction and moment of initiation in trials with and trials without a reversal in movement direction. All tests were performed two-sided with significance level α set to 0.05.

## Results

### Interception Performance

Based on the adopted performance criterium (seat center contacting the 2-m radius target cylinder), participants overall intercepted the target in 78.4% of the trials. Interception performance varied over conditions, with close to maximum performance observed for the Retreat and Cross target directions under the SIDE and CENTER eccentricity conditions (see Table 1). A repeated-measures ANOVA with factors Eccentricity (CENTER, SIDE, SIDE+) and Direction (Approach, Cross, Retreat) revealed significant main effects of the factors Eccentricity [*F*(2,16) = 9.76, *p* = 0.0017, η^2^_p_ = 0.55] and Direction [*F*(2,16) = 18.83, *p* < 0.0001, η^2^_p_ = 0.70], as well as an interaction between the two [*F*(4,32) = 7.05, *p* = 0.0003, η^2^_p_ = 0.47]. *Post hoc* analysis of the overarching interaction demonstrated that under the CENTER condition performance was lower for the Approach direction than for the Retreat and Cross directions (*p*’s < 0.05). While the SIDE condition did not reveal significant effects of target direction, under the SIDE+ condition performance was significantly (*p* < 0.05) lower for the Cross direction than for the Retreat direction. The supplementary analysis revealed no significant differences in performance between Approach and Approach+ directions for either SIDE or SIDE+ eccentricity conditions.

**TABLE 1:** Means and between-participant standard deviations (*M* ± *SD*) of Success Rate (SR), Time until Closest Distance (TCD), and Moment of Initiation of first steering action (MoI) for the Approach+, Approach, Cross and Retreat target directions under the CENTER, SIDE, and SIDE+ target eccentricity conditions.

		SR (%)	TCD (s)	MoI (s)
CENTER	Approach	48.9 ± 32.2	2.85 ± 0.05	0.49 ± 0.03
	Cross	96.7 ± 7.1	3.77 ± 0.04	0.49 ± 0.03
	Retreat	98.9 ± 3.3	4.75 ± 0.03	0.50 ± 0.03
SIDE	Approach+	74.4 ± 13.3	2.25 ± 0.02	0.60 ± 0.06
	Approach	76.7 ± 13.2	2.36 ± 0.01	0.69 ± 0.08
	Cross	94.4 ± 7.3	2.93 ± 0.00	1.19 ± 0.16
	Retreat	98.9 ± 3.3	3.96 ± 0.02	1.04 ± 0.29
SIDE+	Approach+	55.6 ± 16.7	2.22 ± 0.02	0.59 ± 0.05
	Approach	74.4 ± 15.9	2.35 ± 0.01	0.67 ± 0.15
	Cross	60.0 ± 19.6	3.00 ± 0.02	1.06 ± 0.22
	Retreat	83.3 ± 26.0	4.37 ± 0.09	0.89 ± 0.16

*Note that the Approach+ target direction was absent under the CENTER eccentricity condition.*

Notwithstanding these criterium-defined performance differences, participants generally came close to the target on the vast majority of missed trials, as attested to by the overall 0.14-m median closest distance to the target circumference for the 210 missed trials. Subsequent analyses were based on all trials.

### Interception Paths

**FIGURE 3:**
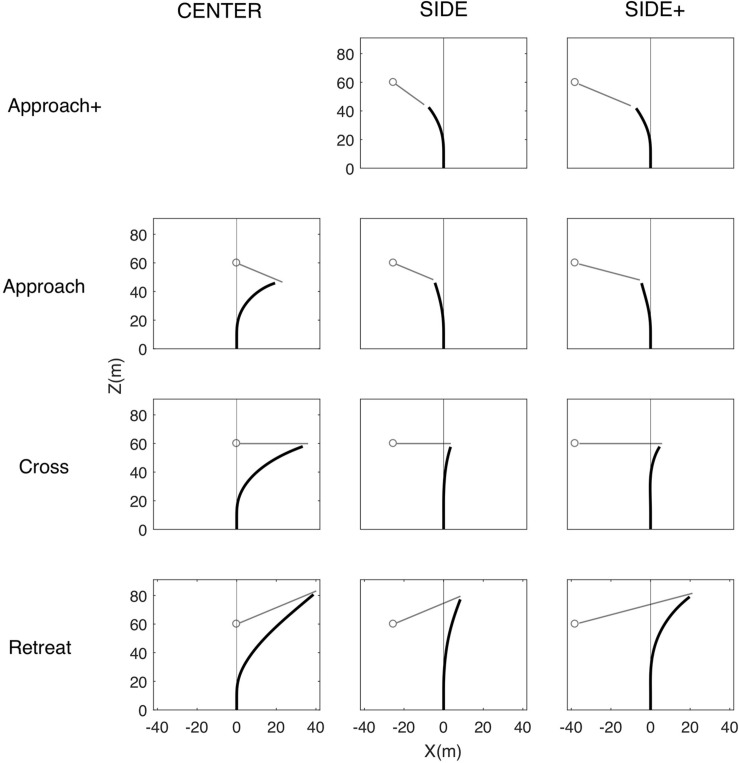
Overall average locomotor paths (in black) under the CENTER, SIDE, and SIDE+ eccentricity conditions for the Approach+, Approach, Cross, and Retreat target directions. Note that the Approach+ target direction was absent under the CENTER eccentricity condition. Gray outline dots and attached line segments indicate initial target positions and subsequent target trajectories.

The (time-averaged) mean interception paths followed by the participants are presented in Figure 3 for all combinations of Target Eccentricity and Target Direction. Inspection of the path shapes under the CENTER and SIDE eccentricity conditions for the Approach, Cross, and Retreat target directions revealed large similarities between our results and those of Fajen and Warren (2004). Interception paths observed under the CENTER condition were characterized by an early and relatively sharp initial turn followed by a straightening out of the interception path, most clearly visible for the Retreat and Cross target directions. Because CENTER conditions caused participants to initially lag the target, the observed straightening out of the path cannot be accounted for by the interception strategy of nulling changes in target-heading angle β. Indeed, such a dβ/dt-nulling strategy cannot explain the sign change in β that necessarily accompanies the change from the initial lag situation (target to the right of participant heading direction, see Figure 3) to the later lead situation (target to the left of participant heading direction). Under the SIDE conditions participants more gradually turned toward the final interception locations, on the left for the Approach direction and on the right for the Cross and Retreat direction. The main difference between Fajen and Warren’s (2004) findings for walking participants and the present findings for driving participants was the absence of a clearly visible S-shaped bend in the initial part of the average interception paths for the Cross and Retreat target directions under the SIDE eccentricity condition. As will become clear further on, however, this result for averaged interception paths does not imply that we did not observe the expected reversals in movement direction at the level of individual trials.

The present study extended the Fajen and Warren (2004) study by (i) inclusion of an additional Approach+ target direction, balancing the distribution of final interceptions on the left and on the right of the participants’ initial heading direction while forcing participants to respond rapidly on certain trials, and (ii) inclusion of an additional SIDE+ target eccentricity in order to evaluate potential effects of initial target eccentricity. As expected, the adaptation (relative to the SIDE eccentricity condition) of target speed and direction under the SIDE+ eccentricity condition gave rise to interceptions at similar locations reached after comparable trial durations (see Table 1) for the Approach+, Approach, and Cross target directions. For the Retreat target direction, these adaptations led to somewhat further outward and later interceptions.

### Moment of Initiation of First Steering Action

The pretrial-onset alignment protocol used in the present study, ascertaining that heading direction was constant (dϕ/dt = 0°/s) at ϕ = 0° at the moment of first target appearance, allowed reliable identification of the moment of first change in heading direction on each individual trial. The earliest steering actions were observed under the CENTER condition where participants initiated steering around 0.5 s after the target appeared for all target directions (see Table 1). In the other Eccentricity conditions, moment of initiation varied with target direction, as confirmed by a repeated-measures ANOVA with factors Eccentricity (CENTER, SIDE, SIDE+) and Direction (Approach, Cross, Retreat) that revealed significant main effects of the factors Eccentricity [*F*(2,16) = 71.60, *p* < 0.0001, η^2^_p_ = 0.90] and Direction [*F*(2,16) = 24.15, *p* < 0.0001, η^2^_p_ = 0.75], as well as an interaction between the two [*F*(4,32) = 13.99, *p* < 0.0001, η^2^_p_ = 0.64].

**FIGURE 4:**
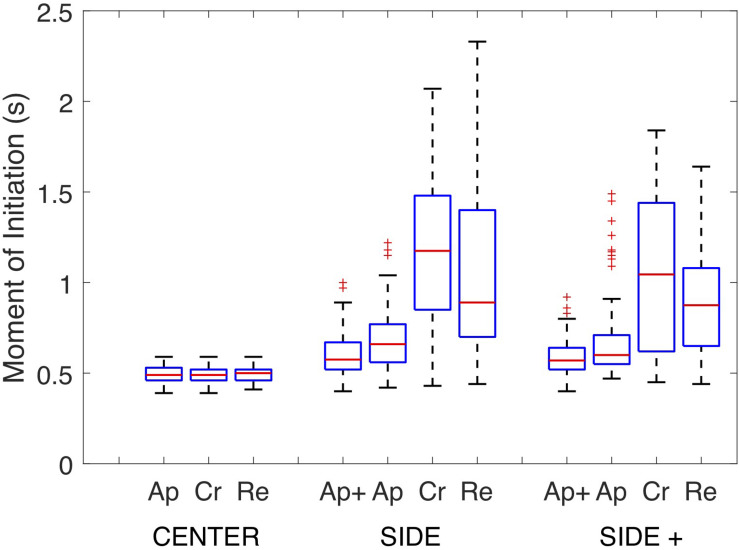
Box plots of the full sets of Moments of Initiation of the first steering action on individual trials under the CENTER, SIDE, and SIDE+ eccentricity conditions for the Approach+ (Ap+), Approach (Ap), Cross (Cr), and Retreat (Re) target directions. Note that the Approach+ target direction was absent under the CENTER eccentricity condition.

*Post hoc* analysis of the overarching interaction demonstrated that under both the SIDE and SIDE+ eccentricity conditions steering action was initiated earlier for the Approach than for the Retreat direction (*p*’s < 0.001) which in turn revealed earlier initiations than for the Cross direction (*p*’s < 0.05). No significant differences in moments of initiation were observed between Approach and Approach+ directions under either SIDE or SIDE+ eccentricity conditions in the supplementary analysis. Figure 4 presents the full set of moments of initiation for all combinations of Target Eccentricity and Target Direction in the form of box plots, revealing the expected larger variabilities in moments of initiation of the first steering action for the Cross and Retreat directions under the SIDE and SIDE+ eccentricity conditions.

### Reversals in Movement Direction

Overall, a reversal in movement direction (RMD) was detected in 130 (i.e., 13.1%) of the total 990 trials. Trials with a RMD were observed for all nine participants, although one participant (P6) only showed three such trials, all occurring within the first block of trials^5^. Importantly, the presence of RMD trials was not randomly distributed over the experimental conditions [χ^2^(10) = 310.34, *p* < 0.0001], indicating that reversals in movement direction generally did not occur accidentally. Indeed, as expected, the grand majority of RMD trials (*n* = 127) occurred when participants were confronted with Cross and Retreat target directions under the SIDE and SIDE+ eccentricities, where targets started from locations to the left and were intercepted at locations to the right of the participants’ initial heading direction. The three remaining RMD trials were observed for the Approach direction, two under the SIDE eccentricity (both by P6 in block 1) and one under SIDE+ eccentricity (by P8 in block 1). No RMD trials at all were observed for the Approach+ target directions under the SIDE and SIDE+ eccentricity conditions, nor for any of the target directions under the CENTER conditions. While the three trials with a RMD observed for the Approach direction all resulted in missing the target, this was generally not the case for the RMD trials observed for the Cross and Retreat conditions under the SIDE and SIDE+ eccentricity conditions. Inspection of success rates for these latter conditions did not bring out clear differences in performance between the trials with and without a RMD (SIDE Retreat: 100.0 vs. 98.7%; SIDE Cross: 90.5 vs. 95.6%; SIDE+ Retreat: 82.9 vs. 83.6%; SIDE+ Cross: 53.5 vs. 71.9%).

Whereas Figure 3 focused on average paths, as analyzed by Fajen and Warren (2004, 2007), Figure 5 presents all individual trial paths for each of the Cross and Retreat target directions under the CENTER, SIDE, and SIDE+ eccentricity conditions. Zooming in on lateral displacement, this figure brings out the co-existence of trials with and without a RMD within the same experimental conditions (i.e., Cross and Retreat target directions under both the SIDE and SIDE+ eccentricity conditions).

**FIGURE 5:**
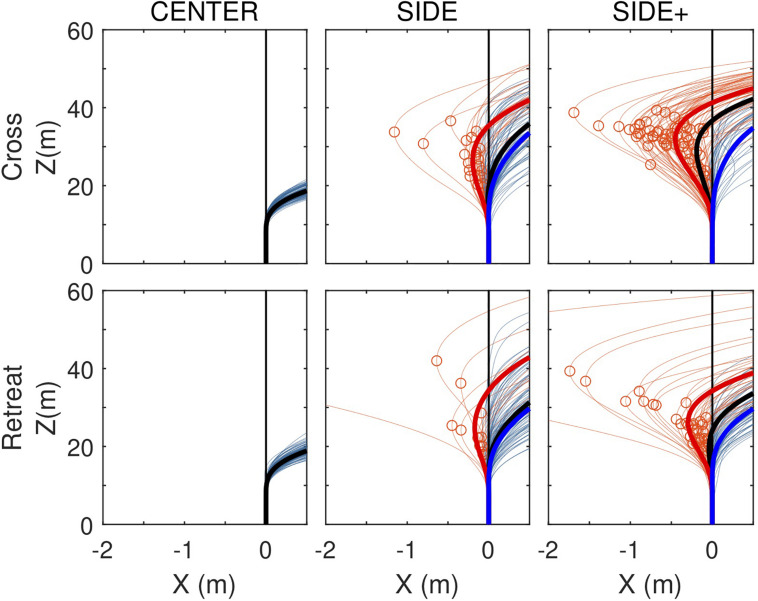
Locomotor paths for individual trials (thin lines) under the CENTER, SIDE, and SIDE+ eccentricity conditions for the Cross and Retreat target directions. Trials with a reversal in movement direction (RMD trials) are depicted in light red and trials without a reversal in movement direction (NoRMD trials) in light blue. Red circles indicate pre-reversal excursion amplitudes. Fat lines represent time-averaged averaged locomotor paths for RMD trials (red), for NoRMD trials RMD (blue), and for all trials (black).

Under the SIDE eccentricity conditions, the Cross and Retreat target directions gave rise to reversals in movement direction in, respectively, 23.3% (*n* = 21) and 14.4% (*n* = 13) of the trials. Under the SIDE+ eccentricity conditions, the Cross and Retreat target directions gave rise to reversals in movement direction in, respectively, 64.4% (*n* = 58) and 38.9% (*n* = 35) of the trials. The number of trials with a RMD was larger [χ^2^(1) = 27.41, *p* < 0.0001] for the SIDE+ eccentricity (*n* = 93) than for the SIDE eccentricity (*n* = 34) and was larger [χ^2^(1) = 7.57, *p* = 0.0059] in the Cross direction (*n* = 79) than in the Retreat direction (*n* = 48).

Not only were trials with a RMD more often observed under the SIDE+ eccentricity than under the SIDE eccentricity for both the Cross and Retreat directions, but for the Cross direction their maximal amplitude was also larger under the SIDE+ eccentricity [0.53 ± 0.34 m vs. 0.25 ± 027 m, *t*(77) = 3.34, *p* = 0.0013; see Figure 5, upper middle and right panels]. Notwithstanding a similar impression from visual inspection of the lower middle and right panels of Figure 5, such a difference could not be demonstrated for the Retreat direction [0.47 ± 0.75 m vs. 0.63 ± 1.51 m, *t*(46) = 0.46, *p* = 0.6420]. Apart from the lower number of trials with a RMD for this target direction, the latter result also appears to be influenced by the presence of a singular large amplitude (but nevertheless successful) RMD trial for the Retreat direction under both the SIDE (4.09 m) and SIDE+ (5.62 m) eccentricity conditions.

Finally, comparison of the trials with a RMD and the trials without a RMD revealed that the former were consistently initiated earlier than the latter (see Table 2). Moreover, within the sets of trials with a RMD, the pre-reversal excursion amplitude was negatively correlated with the moment of initiation of the first steering action, indicating that earlier initiation led to larger excursion amplitudes before heading direction was reversed [SIDE Retreat *r*(33) = −0.483, *p* = 0.0033; SIDE+ Cross: *r*(56) = −0.584, *p* < 0.0001; SIDE Retreat: *r*(11) = −0.354, *p* = 0.2352; SIDE Cross: *r*(19) = −0.504, *p* = 0.0198].

**TABLE 2:** Moments of initiation (MoI, *M* ± *SD*) and statistical comparison *t*-test results of first steering action for trials with a reversal in movement direction (RMD trials) and trials without a reversal in movement direction (NoRMD trials), with number of observations *n*, under the Cross and Retreat direction conditions of the SIDE and SIDE+ eccentricity conditions.

		RMD trials		NoRMD trials			
			
		*n*	MoI (s)	*n*	MoI (s)	*t*(88)	*p*
SIDE	Cross	21	0.99 ± 0.34	69	1.25 ± 0.41	2.62	0.0102
	Retreat	13	0.85 ± 0.31	77	1.08 ± 0.45	1.75	0.0839
SIDE+	Cross	58	0.93 ± 0.40	32	1.29 ± 0.36	4.27	<0.0001
	Retreat	35	0.77 ± 0.27	55	0.96 ± 0.26	3.38	0.0011

## Discussion

The first goal of the present study was to examine whether reversals in movement direction were to be observed in the locomotor behavior of participants steering to intercept targets moving at constant speed along rectilinear trajectories. Based on Fajen and Warren’s (2004) empirical results and simulations of the model proposed by Fajen and Warren (2007), we here expected to observe reversals in movement direction for Cross and Retreat target directions under the SIDE and SIDE+ eccentricity conditions. Although overall average locomotor paths did not reveal clearly visible S-shaped bends (Figure 3), trial-by-trial analysis demonstrated that reversals in movement direction did in fact occur quite frequently under these four specific conditions and almost never under the other conditions of our experiment (Figure 5).

The lack of clearly visible reversals in movement direction in our overall average locomotor paths may have resulted from the task characteristics of turning a steering wheel (continuous, low inertia) to change locomotor direction in the present study rather than modulating bipedal gait (intermittent, high inertia) as in Fajen and Warren’s (2004) study. At the same time, one should also bear in mind that the lack of information on behavior in individual trials in Fajen and Warren’s (2004) results does not allow ascertaining that the overall average behavior they reported was in fact representative of individual-trial behavior.

However, this may be, the present results thus confirmed that reversals in movement direction do indeed occur in an interception-by-steering task with uniformly moving targets, indicating that participants did not uniquely rely on first-order (i.e., rate-of-change based) information. Fajen and Warren’s (2007) suggested that this resulted from the presence of a latency in the detection of target motion, leading participants to initially act as if the target was stationary. Such a latency would moreover allow understanding of the present finding that reversals in movement direction occurred when steering was initiated early on. Initiation of the first steering action early on, presumably before target motion was detected, would indeed give rise to an initial movement in the direction of the target, before reversing direction when its motion was detected and integrated. Initiating a first steering action later on, presumably after target motion was detected, would not give rise to such a reversal in movement direction, also in line with what we observed in the present study. Compared to the SIDE condition, the larger initial target eccentricity of the SIDE+ condition would provide a stronger early drive to move leftward for the Cross and Retreat directions. This fits with our finding of a larger percentage of trials with a reversal in movement direction under the SIDE+ condition than under the SIDE condition for these target directions. It also fits with the finding, at least for the Cross target direction, that trials with a reversal in movement direction had larger pre-reversal excursion amplitudes under the SIDE+ than under the SIDE conditions and that this pre-reversal excursion amplitude was largest for the earliest initiations of first steering action. This, admittedly powerful, latency-based explanation of the observed direction-reversal effect is, however, not without problems, for at least two reasons.

First, our results demonstrated that in trials with a reversal in movement direction steering was initiated between, on average, 0.77 and 0.96 s after target appearance for Cross and Retreat target directions under the SIDE and SIDE+ eccentricity conditions. This implies that the latency explanation of the direction-reversal effect observed in the present study would require a duration of the latency to detect target motion of well over 1 s, more than double the value proposed by Fajen and Warren (2007) for comparable conditions. Moreover, Fajen and Warren (2007) argued that the latency would in fact include both “a visual delay to detect that the target is moving and a locomotor delay to overcome the inertia of the body” (p. 311), both estimated at 0.25 s for walking participants. However, inclusion of a (loco)motor delay is not compatible with the way the latency function actually functions in their model logic: it simply modulates the value of operative dθ/dt (that can become larger or smaller than the real dθ/dt, depending on target motion conditions) and in no way affects the steering dynamics driven by this variable. Put simply, it affects the what and not the how of information used.

Second, this target-motion detection latency cannot explain a related phenomenon: the so-called angle-of-approach effect (Ledouit et al., 2013). This effect also questions unique reliance on first-order information and has been observed in direction-constrained experimental settings for both manual (Peper et al., 1994; Montagne et al., 1999; Dessing et al., 2005, 2009; Jacobs and Michaels, 2006; Michaels et al., 2006; Arzamarski et al., 2007; Dessing and Craig, 2010; Ledouit et al., 2013) and locomotor interception (Bootsma et al., 2016). The angle-of-approach effect refers to the presence of systematic differences in the kinematic patterns of movement when participants intercept constant-speed targets following different rectilinear trajectories that converge onto the same interception location and arrive there after the same target motion duration. In other words, while for different target trajectories, in the end, interception occurs at the same lateral position at the same time, the kinematics (e.g., position and velocity profiles) of the interception movement vary systematically with the target’s angle-of-approach to the common interception location. Given that in Bootsma et al.’s (2016) locomotor interception study participants did not move at trial onset, this angle-of-approach effect cannot be attributed to a flow-parsing induced latency in the detection of target motion.

Overall, we thus now have two apparently robust phenomena questioning unique reliance on first-order information in both manual and locomotor interception of uniformly moving targets: (i) the direction-reversal effect, reported for manual interception by Montagne et al. (1999) and for locomotor interception by Fajen and Warren (2004) and by the present study, as well as (ii) the angle-of-approach effect reported for manual interception in several studies (see Ledouit et al., 2013) and for locomotor interception by Bootsma et al. (2016).

Rather than attributing these effects to an exceptionally long latency in the detection of target motion, even in the absence of self-motion, an alternative explanation would be that for interception of uniformly moving targets participants rely on some kind of combination of angle-related (zeroth-order) and angular rate of change-related (first-order) information, which could take the integrative form of a fractional-order, slightly below 1, as suggested by Bootsma et al. (2016).

We conclude that by carefully designing experimental conditions reversals in movement direction can be evoked in human locomotor interception of uniformly moving targets and that this direction-reversal effect is robust. We also conclude that reversals in movement direction need not consistently occur on all trials of the same experimental condition, as they depend on the timing of the participant’s first action. In the present study, moments of initiation of the first steering action were not only found to vary over conditions in terms of means, but also in terms of distribution, as is clear from inspection of Figure 4. Compared to the other conditions, the relatively late mean moments of initiation observed for the Cross and Retreat target directions under the SIDE and SIDE+ eccentricity conditions were accompanied by fairly large variabilities. Such large variations in the moments of initiation of the first steering action in fact allowed the co-occurrence of trials with a reversal in movement direction and trials without such a reversal in movement direction to come to the fore, as the former were associated with earlier initiations and the latter with later initiations. We suggest that the comparatively large variability in moments of initiation of the first steering action observed for the Cross and Retreat target directions under the SIDE and SIDE+ eccentricity conditions resulted from informational magnitudes remaining close to threshold (for steering action initiation) for an extended period of time under these specific conditions. Even a low degree of noise in the detection of these informational magnitudes would then give rise to the observed variability in timing of steering action initiation and thereby to the emergence of trials with and trials without a reversal in movement direction within the same experimental condition.

## Data Availability Statement

The raw data supporting the conclusions of this article will be made available by the authors, without undue reservation.

## Ethics Statement

The studies involving human participants were reviewed and approved by the Aix-Marseille University Ethics Committee. The patients/participants provided their written informed consent to participate in this study.

## Author Contributions

GC, RC, and RB conceived and designed the study, interpreted the data, and drafted the work. GC and RC implemented and ran the experiment and analyses. RB wrote the final manuscript version that was approved by all authors. All authors contributed to the article and approved the submitted version.

## Conflict of Interest

The authors declare that the research was conducted in the absence of any commercial or financial relationships that could be construed as a potential conflict of interest.

## Footnotes

1 In order to distinguish interception of a moving target as accomplished by means of whole-body displacement from interception as accomplished by hand displacement, we refer to the former as locomotor interception and to the latter as manual interception.

2 Initial conditions were also left/right mirrored, so that participants were in fact confronted with targets moving from the left to the right and from the right to the left. With data being subsequently collapsed, discussion of conditions and results is, for ease of description, based on target motion from left to right.

3 When target motion is not uniform (as in curving trajectories, for instance), the presence of reversals in movement direction does not contradict reliance on first-order information; in particular cases it may then even been taken as evidence in favor of reliance on such first-order information (Dessing and Craig, 2010; Craig et al., 2011).

4 https://ism.univ-amu.fr/en/crvm

5 While P6 showed only three trials with a RMD on the total of 110 trials, the other eight participants showed between 8 and 24 RMD trials each, for an overall *M* ± *SD* of 14.4 ± 6.1 (without P6: 15.9 ± 4.6). RMD trials were observed in comparable numbers over all blocks, with *n* = 34, 26, 19, 27, 24 for blocks 1 to 5, respectively; χ^2^(4) = 4.54, *p* = 0.3380. We note that, of all participants, P6 demonstrated the lowest performance on the task, with P6’s overall average success rate (64.5%) being significantly different [*t*(97), = 2.12, *p* = 0.037] from that of all other participants (80.1%).
